# Recent Progress in Space Physiology and Aging

**DOI:** 10.3389/fphys.2018.01551

**Published:** 2018-11-12

**Authors:** Felice Strollo, Sandro Gentile, Giovanna Strollo, Andrea Mambro, Joan Vernikos

**Affiliations:** ^1^Diabetes Unit, San Raffaele Institute, Rome, Italy; ^2^Campania University “Luigi Vanvitelli” and Nefrocenter Research Network, Naples, Italy; ^3^Endocrinology Unit, FBF San Pietro Hospital, Rome, Italy; ^4^Anesthesiology and Resuscitation Unit, “Misercordia” Hospital, Grosseto, Italy; ^5^Thirdage LLC, Culpeper, VA, United States

**Keywords:** microgravity, aging, space, physiology, translational research

## Abstract

Astronauts coming back from long-term space missions present with different health problems potentially affecting mission performance, involving all functional systems and organs and closely resembling those found in the elderly. This review points out the most recent advances in the literature in areas of expertise in which specific research groups were particularly creative, and as they relate to aging and to possible benefits on Earth for disabled people. The update of new findings and approaches in space research refers especially to neuro-immuno-endocrine-metabolic interactions, optic nerve edema, motion sickness and muscle-tendon-bone interplay and aims at providing the curious - and even possibly naïve young researchers – with a source of inspiration and of creative ideas for translational research.

## Introduction

Astronauts coming back from long-term space missions present with different health problems potentially affecting mission performance and closely resembling those found in the elderly. The latter mostly involve the immune system, bones, muscles, eyes balance and coordination and the cardiovascular system (Strollo, 1999; Vernikos, 2004; Vernikos and Schneider, 2010). The identification of appropriate measures of astronaut performance and of individual genetic or acquired stress resilience features has thus become a research priority along with psychological investigations designed to enhance cognitive performance, sleep, and team-building^1^.

To succeed in such an ambitious task, it is absolutely necessary to start from where we find ourselves today considering that NASA’s 60th anniversary is on October 1, 2018.

We are fully aware that this is not an exhaustive review and should be viewed as a partial synthesis of research findings in this field designed to stimulate the interest of young specialists in the direction we mentioned above. It also is designed to build on earlier reviews rather than repeat them. We will not deal with cardiovascular physiology (Norsk, 2005), which is by far the most studied system in space (Scano and Strollo, 1996), or with pulmonary physiology, an extremely important area where a few groups have done a lot of work (Prisk, 2014), as the reader can easily refer to recent dedicated reviews (Antonutto and di Prampero, 2003). We chose instead to point out the most recent advances in the literature in areas of expertise in which specific research groups were proven to be creative, as well as with respect to aging and to possible benefits on Earth for disabled people.

## Immune Changes and Environmental Stress

Since the very beginning of the space era immune defects were identified in response to real and simulated microgravity (Cogoli, 1993). Many research groups involved in this field confirmed those defects, such as increased virulence, antibiotic resistance, enhanced susceptibility to infections during space missions (Pierson et al., 2005; Stowe et al., 2011). They then explored in greater detail the possible underlying molecular and systemic mechanisms (Sonnenfeld and Miller, 1993). These data were used to identify appropriate countermeasures that might be most beneficial during long-duration missions *per se* (Sonnenfeld et al., 2003) as well as to develop specific preventative exercise protocols that could be performed during the mission (Walsh and Whitham, 2006). These could also be exploited for elderly people on the ground, most of whom undergo some kind of immune deficiency over time as well. Recently, after describing space-related immune system alterations and administering a questionnaire to many experts in the field, the need for longer duration missions devoted to immune research was suggested (Frippiat et al., 2016) along with new space-specific technologies to detect immune changes inflight in minute amounts of blood, reviewed *in situ*, with operational solutions onboard the ISS, with several potential individualized immune countermeasures for exploration missions in the context of precision medicine (Crucian et al., 2018). Animal research studied adult male mice that were exposed to chronic unpredictable mild psychosocial and environmental stressors (CUMS model) for 3 weeks. This period is long enough to simulate in mice a long human flight. This exposure induced an increase of serum IgA, a reduction of normalized splenic mass and reduced production of pro-inflammatory cytokines, as has been previously reported during or after space missions. However, they did not modify either major splenic lymphocyte sub-populations or their proliferative responses, suggesting that the observed inflight changes could be due to other factors such as gravity variations (Gaignier et al., 2018).

## Muscle-Tendon Unit and Exercise as a Countermeasure

Sarcopenia is a typical aging-like effect of microgravity on the muscle-tendon unit structure and function that is still awaiting effective physical, nutritional, or pharmacological countermeasure. These could also be of benefit on Earth for the rehabilitation of injured elderly people. Since the early space flight era, the research in the field has been extensive. Many papers have dealt with the problem of muscle changes (Hargens et al., 2013; Stein, 2013; Ohira et al., 2015). From a nutrition point of view, protein /aminoacid supplementation has been studied as a possible solution that could be exploited also on Earth in inactive elderly people but, in the absence of exercise, no positive effect was obtained in microgravity when increasing either protein or leucine intake (Biolo et al., 2004; Backx et al., 2018). Branched chain amino-acids, however, were found to be somewhat effective, when tested during hind limb suspension in growing rats (Jang et al., 2015) and during bed rest in women (Dorfman et al., 2007).

After many years of poor results obtained with the exercise programs routinely carried out onboard the international space station, a possible solution may be provided by the horizontal sled jump method (Kramer et al., 2017, 2018) tested in Cologne during an ESA Bed Rest study. A similar device might be adapted in the future in terms of time/intensity of muscle involvement, to prevent sarcopenia in temporarily bed-ridden, non-frail elderly people (Vernikos, 2017). This “SPRINT” protocol of repeated short duration sessions of different kinds of high intensity aerobic and resistance exercise was deemed to represent both a promising inflight multi-system countermeasure and a potentially viable strategy for pre-habilitation before elective medical procedures on Earth (Ploutz-Snyder et al., 2018). However, compared with resistance training, whole-body vibration (WBV) is safer and more convenient while avoiding the risk of injury in older adults. A recent study using 12 Hz – 3 mm WBV (10 repetitions, each lasting 60 s, followed by a 30 s resting period per session, 3 times a week for 3 months) showed significantly positive effects on muscle mass, physical fitness (standing on one foot, flexibility, sit-to-stand, and grip strength) in sarcopenic institutionalized elderly people (Chang et al., 2018).

As far as basic mechanisms are concerned, unloading/disuse sarcopenia is still poorly understood and should be further investigated. Effective signal manipulation methods are needed that are applicable to muscle wasting prevention in the elderly. In mice. mechanical unloading reduced the expression of irisin, a muscle-derived insulin-sensitizing and bone-enhancing cytokine, presumably through pathways involving osteocalcin, a bone morphogenic protein (BMP), and PI3K suggesting that irisin might be involved in muscle/bone relationships regulated by mechanical stress (Kawao et al., 2018). Tendon physiology still awaits greater understanding. Appropriate training should be taken into account in preventing unexplained injuries especially in elderly people trying to get fit after decades of sedentary life (Magnusson and Kjaer, 2018).

## Insulin Resistance and Endocrine Changes

Insulin sensitivity changes that typically accompany aging on Earth and that gradually may progress to diabetes (a condition of decompensated insulin resistance) have been repeatedly reported as a consequence of space flight (Tobin et al., 2002; Hughson et al., 2016) and bed rest (Bergouignan et al., 2011) and assumed to be partly due to the cephalad fluid shift but especially dependent on unloading-related sarcopenia. Based on cross-sectional intervention studies (Lillioja et al., 1987; Prior et al., 2015), reduced muscle capillarization was thought to be strongly involved in metabolic disturbance (Stuart et al., 1988). However, a paper published more recently, clearly showed that after only 4 days of bed rest both insulin sensitivity and skeletal muscle fiber cross-sectional area decreased, while capillary density increased, in healthy young subjects (Montero et al., 2018). This suggests that muscle capillaries do not primarily influence insulin sensitivity through impaired nutrient delivery in the absence of aging or pathological conditions (Montero et al., 2016). The most relevant changes responsible for insulin resistance might involve the expression and/or activity of insulin receptor β-subunit, Akt, GLUT4, AMPK and many other intracellular signals known to be involved in the insulin effect (Lassiter et al., 2018). Eventually, liver dysfunction may further contribute to insulin-resistance during long duration missions (Jonscher et al., 2016; Gentile et al., 2016). Since the beginning of manned flights other possible endocrine defects involved in aging have also been investigated in space but uncertainties remain, probably due to the fact that all of them are extremely sensitive to the stress experienced by astronauts both in flight and upon re-entry. Therefore, many of the observed changes can reflect other factors rather than the effects of microgravity *per se*. This especially applies to fluid-electrolyte regulating hormones and the glucoactive adrenal hormones, cortisol/corticosterone, which continue to be reported as increased in flight by most authors (Macho et al., 2001) despite some conflicting results (Grindeland et al., 1990; Strollo, 2000). As for thyroid hormones, production was reported to be impaired in rats and monkeys kept at a low temperature for 2 days after a 12-day Cosmos 1887 flight and rat thyroid hyperactivity was reported up to 14 days after re-entry (Plakhuta-Plakutina et al., 1990), This led to the hypothesis of a typical microgravity-induced hypothyroid state with post-flight rebound, further supported by the increased TSH levels found in humans (Leach et al., 1977). In fact, this was contradicted when iodine was no longer added to drinking water for hygienic purposes as after the Spacelab 2 flight. Blunted thyroid C-cell calcitonin secretion (Plakhuta-Plakutina et al., 1988) and enhanced PTH secretion by the parathyroid glands (Plakhuta-Plakutina, 1979) were also reported in earlier flights. Testosterone was measured occasionally in male astronauts (Smith et al., 2012) and more often in bed-rest conditions in healthy volunteers (Wade et al., 2005). Carefully designed dedicated studies are still needed to resolve conflicting results (Strollo et al., 1998b), that often show no changes in the human in bed-rest but point to possible negative effects not only in inflight tests on astronauts but also in experiments carried out in flight and on ground using cellular or animal models (Amann et al., 1992; Uva et al., 2005). Much more would be learned from future inflight endocrine signal measurements, which will require conditions and equipment specifically designed for space, such as miniaturization, use of dry chemistry methods or minimization of liquid handling procedures and quality control assurance of laboratory equipment analysers.

## Osteoporosis

A well-known negative consequence of the unloading in spaceflight is osteoporosis which also affects most of the geriatric population. Their study has been addressed by leading groups in the field since the very beginning of the space era. We encourage young readers to study thoroughly the original work (Morey-Holton and Globus, 1998; Alexandre and Vico, 2011; Cappellesso et al., 2015). With respect particularly to postflight rehabilitation needs, a very interesting finding is worth mentioning: loading during the early post-fracture stages, while matrix deposition and remodeling are prevalent, may enhance repair through the formation of additional cartilage and bone (Liu et al., 2018). In fact, not only bone but also cartilage problems have been reported in microgravity. These include smaller chondrogenic pellets, less proteoglycan synthesis and reduced dynamic stiffness of three-dimensional engineered cartilage constructs (Freed et al., 1997). These observations warrant further investigation because synovial joints, which contribute to articular cartilage, subchondral bone, meniscal fibro-cartilage, tendon and ligaments bathed in synovial fluid and enclosed in a fibrous capsule, may undergo damage, especially upon reloading on return to Earth Joint instability and compensatory connective tissue changes resulting in joint degradation would follow (Fitzgerald, 2017).

Space osteoporosis is characterized by a lower bone formation rate and enhanced bone resorption, which calls for careful and timely preventative strategies, including Vitamin D_3_ and K_2_ supplementation and antiresorptive drug administration (Iwamoto et al., 2005). Bisphosphonates are the most commonly used anti-bone-resorptive drugs on Earth. Although they have also been used to prevent space-related osteoporosis, the results are conflicting (Cavanagh et al., 2005). The reason for this depends on the fact that bone formation is coupled to bone resorption and therefore about one month after antiresorptive effects begin, a “frozen” bone remodeling condition occurs (Bauer et al., 2018). This seems to be particularly the case during unloading. However, as significant increases in bone microarchitecture can be achieved with low-dose anti-resorptive therapy without reducing bone formation, such an anti-resorptive dosing approach could be used to preserve bone quality by limiting it to the period of disuse. This could provide astronauts with faster recovery and less adverse effects (Lloyd et al., 2008). Meanwhile, the current combination of bisphosphonates with exercise (Leblanc et al., 2013) seems to be the accepted compromise.

## Melatonin and Sleep-Unrelated Functions

A progressive decrease in night-time melatonin secretion is observed during aging accompanied by and often causing severe sleep disturbances (Bubenik and Konturek, 2011; Duffy et al., 2015). A similar reduction in melatonin has been reported in microgravity (Holley et al., 1991). Night-time and day-time and sleep are disrupted in space flight by a variety of factors, including circadian rhythm disturbances due to the added effects of continuous exposure to office-like dim light, sleep-disrupting noise levels in the spacecraft during rest and 90’ day/night, light /dark cycles through the spaceship windows (Dijk et al., 2001). Furthermore, cosmonaut Atkov reported the discomforting absence of the sensation of putting the weight of his head down on a pillow in microgravity (Vernikos, personal communication). Others report although they seem to have slept enough hours they do not feel rested when they wake up. All of these may be responsible for disturbing normal variations in sleep-wake markers and therefore sleep and circadian rhythms need to be explored systematically both on the ground and in space, in humans and non-humans, for the sake of healthy sleep and consequent work efficiency and for better quality of life.

Great strides have been made in the last decade in the field of sleep, sleep deprivation and the body’s timing mechanisms as to its relationship to the environment, that have not been applied to space.

Related to this a recent paper was published though apparently dealing with something else: the bone resorption marker amino-terminal cross-linked collagen I telopeptide (NTx) was found to be increased in 20 premenopausal women volunteering for continuous wake and dim light conditions as compared to normal environmental conditions, thus confirming both the existence of an endogenous circadian rhythm in NTx with a night-time peak and the synchronizing influence of environmental factors on it (St Hilaire et al., 2017). Lower bone mineral density (Kim et al., 2013) and even increased risk of hip and wrist fractures (Feskanich et al., 2009) have been reported in shift workers who are exposed to chronic circadian, sleep and melatonin disruption. Similar effects, though maybe only additive to the already known role of muscle and bone unloading, may be expected for others with chronic circadian misalignment, such as astronauts. These should be taken into account when dealing with integrative physiology, which in fact often opens unexpected connecting paths among seemingly unrelated systems.

## Cns Changes

Cognition and behavior may be impaired in microgravity due to changes in cerebrovascular circulation including the increased blood pressure caused by the redistribution of body fluid (Strollo et al., 1998a; Lakin et al., 2007). The anatomical configuration of the brain and cerebrospinal fluid (CSF) spaces were found to change as a consequence of long term space flight: magnetic resonance imaging (MRI) studies showed narrowing of the central sulcus, upward shift of the brain, and narrowing of CSF spaces at the vertex in most astronauts studied (Roberts et al., 2017; Van Ombergen et al., 2017b). The duration and clinical significance of such findings warrants further systematic study of previously observed anatomical brain changes during bed rest and evidence from functional MRI (fMRI) signs of performance related cortical reorganization (Roberts et al., 2010; Roberts et al., 2015). This may be relevant to vestibular patients and elderly people with multisensory deficit syndromes or in merely inactive individuals (Van Ombergen et al., 2017a).

Most pertinent functional studies concentrated on spatial orientation, object recognition, motion perception and high-level cognitive functions including learning, memory, reasoning and calculation (Kornilova, 1997; Leone, 1998; Koga, 2000; Shehab and Schlegel, 2000; Kelly et al., 2005). To investigate this further a 7-day head-down tilt (-6° bedrest) study involving 20 subjects concluded that the first three days in space should be considered as potentially critical for cognitive performance as based on mental rotation tests (Wang et al., 2017).

Postural stability is yet another aspect of CNS performance that is affected both by spaceflight as well as aging (Demertzi et al., 2016). Increased cortico-spinal drive from leg motor cortex to lower limb motor neurons was found following postural perturbations in the elderly along with impaired perceptual processing of sensory afferent signals, which form the basis of prolonged muscle response delays during perturbed balance (Ozdemir et al., 2018). A close association between neural and muscular factors for morphological and functional adaptation to space flight was found (Ohira, 2000). This led to the proposed theory that the whole postural control system is tied together by links between vestibular, visual and somatosensory information. On Earth, these develop and are kept spontaneously active through experience of inertial and gravitational reaction forces, while in microgravity they should be actively established for postural and perceptual stability (Mergner and Rosemeier, 1998). Not to mention lunar or planetary microgravity environments where gravity levels may be at or below sensory thresholds. According to this concept, biomechanics and multi-body dynamics should be taken into account, as well as, the feedback and possibly feed forward loops used for postural control, whose complexity may be impossible to resolve in the absence of current developments in computer science and robotics. To resolve such a sophisticated puzzle as postural control may still be far from being achieved. Precise mechanisms implemented by the brain on a neural or molecular level will probably not be achieved any time soon even with the most powerful calculation tools available.

## Optic Nerve Edema

Closely linked to changes occurring in the CNS, a threatening and still poorly managed side-effect of long duration space flight is optic nerve head (ONH) edema causing reduced visual acuity with both health and mission consequences. The overall eye defects range from changes in refractive error and varying degrees of disk edema to globe flattening, choroidal folds, and cotton-wool spots (Mader et al., 2011; Taibbi et al., 2013) and are referred to as space flight-associated neuro-ocular syndrome (SANS) correlated with intra-orbital and intracranial magnetic resonance imaging both in-flight, as well as, in terrestrial ultrasonographic and ocular optical coherence tomography findings (Lee et al., 2017). How this happens is unknown and yet to be determined. It seems to be at least partly explained by increased intracranial pressure accompanying the cephalad fluid shift, venous outflow obstruction, blood-brain barrier breakdown, and disruption in CSF flow with local effects on ocular structures (Wiener, 2012), individual differences in metabolism, and the vasodilator effects of carbon dioxide (Michael, 2018). A suitable treatment of ONH depends on a better understanding of the underlying mechanisms with research moving more in this direction. Using pre- and post-flight optical coherence tomographic scans of the ONH region, global and quadrant total retinal thickness, retinal nerve fiber layer (RNFL) thickness and choroidal thickness have been calculated: circumpapillary RNFL thickness was found to be increased by a median of 2.9 μm without any change in choroidal thickness. This imaging technique is now expected to allow the assessment of longitudinal changes and the development and testing of countermeasures in astronauts, as well as potentially in patients suffering from disk edema on Earth (Patel et al., 2018).

## Vestibular Defects and Motion Sickness

Another typical problem experienced by the elderly, as well as by astronauts and cosmonauts, especially at the beginning of their space flight, is motion sickness. Selection of candidates to minimize the risk has always been the preferred solution, nevertheless training procedures have also been investigated throughout the space program. Allocating less attention to the central field during visual motion stimulation has been recently found to be associated with more stable vection and higher resistance to motion sickness (Wei et al., 2018). Virtual reality application designers may use this finding to strengthen and stabilize vection, while reducing the risk of visually induced motion sickness.

On the other hand, impaired vestibular function in the elderly can result in worsened gaze stabilization with an increased risk of falling. Ocular-counterroll (OCR), a gaze-stabilizing mechanism involving ocular torsion in a compensatory direction to lateral roll-tilt of the head, is predominantly mediated by the otoliths at low frequencies of motion and has been shown to be a sensitive marker of such a defect. In fact, a reduction in OCR gain with aging correlates with increased postural sway, suggesting OCR loss may also be a useful predictor of fall risk (Serrador et al., 2009). A recent paper showed that low levels of subcutaneous electrical stochastic noise (SN), known to enhance weaker signals in nonlinear systems through the phenomenon of resonance, improves otolith-ocular function in aging people with impaired otolith responses without inducing hypersensitivity or other adverse effects in those with normal function. Electrical SN may then represent an effective and well-tolerated non-invasive alternative or add-on to existing rehabilitation strategies. Moreover, its noise-based mechanism is expected to require no adaptation by neural systems, thus theoretically making long-term treatment more feasible (Serrador et al., 2018). Another possible solution may be electro-acupuncture (EA), a method of applying bilaterally, an electric current by means of a special device to needles fitted to specific points, i.e., PC 6 (Pericardium 6), ST 36 (Stomach 36), and LI 4 (Large Intestine 4) at least half an hour before the exposure to disrupting vestibular stimuli (Fydanaki et al., 2017).

## Space Anemia

Anemia in space is also a typical aging-like consequence of microgravity requiring greater understanding not only for the sake of astronauts but also to improve the quality of life of millions of elderly people all around the world (Rizzo et al., 2012). In the early days if spaceflight it was believed to be a consequence of exposure to the high environmental oxygen concentrations required to compensate for low cabin pressure, and after the normalization of intra-vehicular air pressure/composition, it was hypothesized to reflect either “acute plethora” of red blood cells resulting from fluid shifts or bone calcium reabsorption dependent increased oxygen displacement from circulating hemoglobin (Hughes-Fulford, 1993; De Santo et al., 2005). Now that these mechanisms have been ruled out, anemia is believed to depend on compensatory blunted erythropoietin-release in response to decreased intravascular plasma volume with consequent hemo-concentration and/or enhanced haemolysis (Tavassoli, 1982; Hargens et al., 2013; Kunz et al., 2017). Actually, the results from a specifically designed head down bed rest study do not seem to support such a mechanism since circulating markers of haemolysis did not change throughout the test despite hemoconcentration (Trudel et al., 2017). This may be of high clinical significance since erythrocyte loss from prolonged bed rest partly explains the unpredictable anemia in bed-ridden patients or in people with decreased mobility (Guralnik et al., 2004), an extremely prevalent defect in the elderly population. An often-ignored finding that warrants further investigation is that erythrocytes recover four weeks after the bed rest-induced, decreased production rather than increased haemolysis (Ryan et al., 2016).

## Pharmaco-Kinetics and –Dynamics

Aging often alters pharmacokinetics and pharmacodynamics of life-saving drugs. Similarly, microgravity induces many physiological adaptations and therefore pharmacology in space is a work in progress because it requires continuous monitoring of the physiological changes and pharmacological behavior incrementally with each successive mission duration.

Besides possible conformational receptor-drug interaction changes and metabolic clearance rate at single organ levels, numerous factors unrelated to microgravity, may affect drug effects and thus interfere with normal physiology (Vernikos, 2018, in press):

• abnormal radiation or CO2 levels;
• sources of chemical sterilization of water, such as iodine, which was used for many years to sterilize the water in the United States program and was found to have toxic actions;
• spacecraft light intensity is below threshold for maintaining metabolic biological rhythms;
• day-night cycles in earth orbit last 90 min, and are not coordinated with light/dark and work cycles either inside the spacecraft or with ground control communications;
• occasionally missions operate on a shift-work basis further complicating circadian rhythm issues;
• activities such as EVAs, daily exercise, work-load, eating, social interaction, participation of astronauts as test-subjects in flight experiments or drugs administered as preventive or countermeasures.

Nasal route drug administration for local and systemic delivery of many drugs is an attractive strategy for clinicians as the nasal cavity is highly vascularized and provides a large surface area for drug absorption. It is especially good when targeting the brain because neural pathways such as the olfactory and trigeminal nerves may be directly accessed. However, polar molecules cannot cross the nasal mucosa, and therefore effective solutions must be devised to enable them to cross the blood-brain barrier. Actually *in vitro* studies showed that hypergravity may be an effective new strategy for that. Such a finding might be of great interest in space pharmacology (Vernikos, in press) where enhanced nasal drug delivery in microgravity may be desired (Kim et al., 2018).

## 3D-Scaffolds of Stem Cell Derived Multicellular Spheroids

Both drug research and surgery nowadays largely rely on synthetic tissues: more and more organs fail as age increases and transplantations suffer from shortage of donors all over the world while bioethics asks more and more for cruelty-free drug research. That’s why for the last ten years or so artificial organs have become the object of extensive research, which seems to pave the way to productive large-scale solutions. For a long time, the development of three-dimensional (3D) structures, so-called multicellular spheroids (MCSs), was of great interest to those wishing to investigate various biological processes such as growth, proliferation, differentiation, and drug effects beyond the limits imposed by isolated cells and even single-layer cell biology. The most important finding was that MCSs could be easily produced within hours when growing cells in the Rotating Wall Vessel (RWV) or the Random Positioning Machine (RPM). These devices which allow investigators to perform more and more fruitful experiments mimic the effects of microgravity on Earth providing a useful alternative to actual microgravity facilities like those provided by the KUBIK hardware initially and the BIOLAB onboard the International Space Station (ISS) thereafter, as well as, by Russian retrievable “Bion” or “Foton” satellites, followed by smaller ones based on the CubeSat standard or combining several cubes together (e.g., the United States “PharmaSat-1,” the “GeneSat-1,” or the “SporeSat”). Original stem cells and those derived from human umbilical cord blood progenitors are more and more widely used to engineer 3D constructs under low-gravity conditions. They represent ideal tools for Regenerative Medicine since, in contrast to mature cells, they are able to differentiate into several different cell types, thus allowing the production of a large variety of patient autologous tissues, can be cultured for a very long time while keeping costs low, by exploiting simulated microgravity without actually going into space (Grimm et al., 2018).

## EVA and Decompression Sickness (DCS) Risk

Decompression syndrome and altitude sickness were thought to affect only the young but nowadays more and more middle-aged people progressively becoming old and specific sports-naïve elderly people decide to spend their money in a different way than before to test their performance limits and feel younger than they are by getting involved in underwater or mountain activities. Although this might seem different from what happens in flight, space can be viewed as a suitable, yet involuntary, test bed for possible life-threatening events faced by elderly people on Earth. Before the very first EVA (extravehicular activity) was carried out NASA realized that DCS due to variable degrees of venous and arterial nitrogen embolism either limited to tendons (causing “bends”) or generalized (lung and brain lesions) was a risk that had to be mitigated. The current NASA space-suit referred to as the EMU (extravehicular mobility unit) operates at 4.3 psia or 222 mm Hg above the vacuum of space whereas the Russian Orlan space-suit operates at 5.8 psia. Increasing the space-suit pressure and/or reducing cabin inert pressure are the two ways to decrease the pressure gradient between environments to help minimize risk of DCS as complete elimination of DCS is practically impossible. However, despite these concerns, there have been no recorded cases of DCS in astronauts in pressurized space-suits during EVA, in contrast to altitude chamber technicians, who are much more prone to symptoms or signs of DCS. This might be explained by potential operational and gravitational benefits of the spaceflight upward fluid shift and pre-breathing exercise that induce faster denitrogenation (Sherman and Sladky, 2018).

## Conclusion

Readers looking for a systematically organized, exhaustive review of space physiology may be disappointed. Numerous such reviews have been published by these and other authors. However, due to the relentless development of science, such reviews run the risk of soon being outdated whatever the scientific relevance of the subject and competence of the authors.

As stated in the introduction, our intention was to offer the reader an update of new findings and translational approaches in space research, as advanced techniques and theories become available and the cross-talk between Space, Earth and Aging physiology continues to enrich scientific and social relevance. We hope to have provided the curious – and even possibly naïve young researchers – with a source of inspiration and of creative ideas.

## Author Contributions

FS and JV wrote the paper based on their longstanding space research experience. SG provided the group with his experienced support with respect to issues related to metabolism and internal medicine. GS provided the group with her experienced support with respect to issues related to geriatrics and endocrinology. AM realized a thorough search of the literature and supported the group in the fields of sleep, decompression sickness and EVA.

## Conflict of Interest Statement

The authors declare that the research was conducted in the absence of any commercial or financial relationships that could be construed as a potential conflict of interest.

## Footnotes

1 A Midterm Assessment of Implementation of the Decadal Survey on Life and Physical Sciences Research at NASA; http://nap.edu/24966. Last accessed July 24, 2018.
